# Marvels of Man

**Published:** 1877-04

**Authors:** 


### HALL'S JOURNAL OF HEALTH.

**Our Legitimate Scope is almost boundless: for whatever begets pleasurable and harmless feelings, promotes Health; and whatever induces disagreeable sensations, engenders Disease.**

WE AIM TO SHOW HOW DISEASE MAY BE AVOIDED, AND THAT IT IS BEST, WHEN SICKNESS COMES, TO TAKE NO MEDICINE WITHOUT CONSULTING A PHYSICIAN.

**Vol. 24.** APRIL, 1877. [**No. 4.**

### MARVELS OF MAN.

WHILE the gastric juice has a mild, bland, sweetish taste, it possesses the power of dissolving the hardest food that can be swallowed; it has no influence whatever on the soft and delicate fibers of the living stomach, nor upon the living hand, but, at the moment of death, it begins to eat them away with the power of the strongest acids.

There is dust on sea, on land; in the valley, and on the mountain-top; there is dust always and every where; the atmosphere is full of it; it penetrates the noisome dungeon, and visits the deepest, darkest caves of the earth; no palace-door can shut it out, no drawer so "secret" as to escape its presence; every breath of wind dashes it upon the open eye, and yet that eye is not blinded, because there is a fountain of the blandest fluid in nature incessantly emptying itself under the eyelid, which spreads it over the surface of the ball at every winking, and washes every atom of dust away. But this liquid, so mild, and so well adapted to the eye itself, has some acridity, which, under certain circumstances, becomes so decided as to be scalding to the skin, and would rot away the eyelids were it not that along the edges of them there are little oil manufactories, which spread over their surface a coating as impervious to the liquids necessary for keeping the eye-ball washed clean, as the best varnish is impervious to water.

The breath which leaves the lungs has been so perfectly divested of its life-giving properties, that to rebreathe it, unmixed with other air, the moment it escapes from the mouth, would cause immediate death by suffocation; while if it hovered about us, a more or less destructive influence over health and life would be occasioned; but it is made of a nature so much lighter than the common air, that the instant it escapes the lips and nostrils, it ascends to the higher regions, above the breathing-point, there to be rectified, renovated, and sent back again, replete with purity and life. How rapidly it ascends, is beautifully exhibited any frosty morning.

But foul and deadly as the expired air is, Nature, wisely economical in all her works and ways, turns it to good account in its outward passage through the organs of voice, and makes of it the whisper of love, the soft words of affection, the tender tones of human sympathy, the sweetest strains of ravishing music, the persuasive eloquence of the finished orator.

If a well-made man be extended on the ground, his arms at right angles with the body, a circle, making the navel its center, will just take in the head, the finger-ends, and feet.

The distance from "top to toe" is precisely the same as that between the tips of the fingers when the arms are extended.

The length of the body is just six times that of the foot; while the distance from the edge of the hair on the forehead, to the end of the chin, is one tenth the length of the whole stature.

Of the sixty-two primary elements known in nature, only eighteen are found in the human body, and of these, seven are metallic. Iron is found in the blood; phosphorus in the brain; limestone in the bile; lime in the bones, dust and ashes in all! Not only these eighteen human elements, but the whole sixty-two, of which the universe is made, have their essential basis in the four substances—oxygen, hydrogen, nitrogen, and carbon, representing the more familiar names of fire, water, saltpeter, and charcoal; and such is man, the lord of earth! a spark of fire, a drop of water, a grain of gunpowder, an atom of charcoal! But looking at him in another direction, these elements shadow forth the higher qualities of a diviner nature, of an immortal existence. In that spark is the caloric which speaks of irrepressible activity; in that drop is the water which speaks of purity; in that grain is the force by which he subdues all things to himself, makes the wide creation the supplier of his wants, and the servitor of his pleasure; while in that atom of charcoal, there is the diamond, which speaks at once of light and purity, of indestructibility and of resistless progress, for there is nothing which outshines it; it is purer than the dew-drop; "moth and rust corrupt" it not, nor can ordinary fires destroy; while it cuts its way alike through brass and adamant and hardest steel. In that light we see an eternal progression towards omniscience; in that purity, the goodness of a divine nature; in that indestructibility, an immortal existence.

